# Exploring the Expression of CD73 in Lung Adenocarcinoma with *EGFR* Genomic Alterations

**DOI:** 10.3390/cancers17061034

**Published:** 2025-03-20

**Authors:** Elodie Long-Mira, Christophe Bontoux, Guylène Rignol, Véronique Hofman, Sandra Lassalle, Jonathan Benzaquen, Jacques Boutros, Salomé Lalvée-Moret, Katia Zahaf, Virginie Lespinet-Fabre, Olivier Bordone, Sophia Maistre, Christelle Bonnetaud, Charlotte Cohen, Jean-Philippe Berthet, Charles-Hugo Marquette, Valerie Vouret-Craviari, Marius Ilié, Paul Hofman

**Affiliations:** 1 Laboratory of Clinical and Experimental Pathology, IHU RespirERA, Biobank Côte d’Azur BB-0033-00025, FHU OncoAge, Centre Hospitalier Universitaire de Nice, 06000 Nice, France; long-mira.e@chu-nice.fr (E.L.-M.); bontoux.christophe@iuct-oncopole.fr (C.B.); rignol.g@chu-nice.fr (G.R.); hofman.v@chu-nice.fr (V.H.); lassalle.s@chu-nice.fr (S.L.); ilie.m@chu-nice.fr (M.I.); 2 Institute for Research on Cancer and Aging, Team 4, Inserm U1081, CNRS UMR 7413, Université Côte d’Azur, 06000 Nice, France; valerie.vouret@unice.fr; 3 Department of Thoracic Oncology, IHU RespirERA Hôpital Pasteur, Centre Hospitalier Universitaire de Nice, Université Côte d’Azur, 06100 Nice, France; benzaquen.j@chu-nice.fr (J.B.); boutros.j@chu-nice.fr (J.B.); marquette.c@chu-nice.fr (C.-H.M.); 4 Department of Thoracic Surgery, Hôpital Pasteur, Centre Hospitalier Universitaire de Nice, Université Côte d’Azur, 06100 Nice, France; cohen.c@chu-nice.fr (C.C.); berthet.jp@chu-nice.fr (J.-P.B.)

**Keywords:** non-small cell lung cancer, lung adenocarcinoma, CD73, PD-L1, immunotherapy, *EGFR*, immunohistochemistry

## Abstract

Background/Objectives: Immune checkpoint inhibitors (ICIs) benefit some lung cancer patients, but their efficacy is limited in advanced lung adenocarcinoma (LUAD) with *EGFR* mutations (*EGFRm*), largely due to a non-immunogenic tumour microenvironment (TME). Furthermore, *EGFRm* LUAD patients often experience increased toxicity with ICIs. CD73, an ectonucleotidase involved in adenosine production, promotes tumour immune evasion and could represent a novel therapeutic target. This study investigates CD73 expression in LUAD with *EGFR* alterations and its clinico-pathological correlations. Methods: CD73 expression in tumour (CD73_TC_) and stromal (CD73_SC_) cells was assessed in 76 treatment-naive LUAD patients using immunohistochemistry (IHC) (D7F9A clone) alongside IHC PD-L1 (22C3 clone). *EGFR* alterations were identified by molecular sequencing and FISH. Event-free survival (EFS) was analysed based on CD73_TC_ expression. Results: CD73_TC_ expression was observed in 66% of cases, with high expression (Tumour Proportion Score > 50%) correlating with improved EFS (*p* = 0.045). CD73_TC_ and PD-L1 expression were not significantly correlated (*p* = 0.44), although a weak inverse trend was observed. CD73_SC_ expression was detected in 18% of cases, predominantly in early-stage (*p* = 0.037), PD-L1-negative (*p* = 0.030), and non-*EGFR*-amplified (*p* = 0.0018) tumours. No significant associations were found with disease stage, histological subtype, *EGFR* mutation type, and amplification. Conclusions: CD73 expression in *EGFRm* LUAD is heterogeneous and associated with diverse TME profiles. These findings support the potential of CD73 as a predictive biomarker and therapeutic target, highlighting its clinical relevance in *EGFRm* LUAD.

## Simple Summary

Lung cancer patients with *EGFR* mutations experience limited benefits from immune checkpoint inhibitors due to a tumour microenvironment that hinders the immune response. CD73, a protein involved in immune evasion, has emerged as a new promising therapeutic target. This study evaluates CD73 expression in tumour and stromal cells of lung adenocarcinoma with *EGFR* genomic alteration and explores its association with clinical and molecular features. Our findings show that high CD73 expression in tumour cells correlates with longer event-free survival and a unique immune environment characterised by low PD-L1 expression. These results suggest that CD73 could serve as both a predictive biomarker and a therapeutic target, potentially guiding the development of new treatments for *EGFR*-mutated lung cancer.

## 1. Introduction

Non-small cell lung carcinoma (NSCLC) remains one of the most challenging forms of cancer to treat, particularly in the context of *EGFR* mutations. The advent of third-generation tyrosine kinase inhibitors (TKIs) has significantly improved the management of *EGFR*-mutated (*EGFRm*) lung adenocarcinoma (LUAD), establishing these inhibitors as the standard of care for first-line treatment [1,2,3]. However, various mechanisms of resistance to EGFR TKIs poses significant clinical challenges. Primary resistance occurs in 10 to 30% of cases [4,5] and is influenced by factors such as the type of *EGFR* mutation [6,7], mutant allele frequency [8], co-occurring genetic alterations [9,10,11,12], and the immunosuppressive tumour microenvironment [13]. Moreover, all patients with *EGFR* mutations will experience acquired resistance to TKIs, limiting the long-term effectiveness of these treatments [14,15,16]. One of the mechanisms contributing to resistance to EGFR TKIs, notably at diagnosis, is the amplification of the *EGFR* gene (*EGFR-amp*), leading to increased protein expression and activation of downstream signalling pathways that promote tumour growth and survival [17]. An increased *EGFR* gene copy number has been reported in 50–75% of LUAD cases with *EGFR* mutations either before or after treatment [18,19,20]. 

While immune checkpoint inhibitors (ICIs), targeting PD-1 (programmed cell death protein 1), PD-L1 (Programmed Death-Ligand 1), and CTLA-4 (cytotoxic T-lymphocyte antigen-4), have become standard care for advanced NSCLC without actionable mutations [21], their effectiveness in *EGFRm* NSCLC is limited, with low response rates [22,23,24,25], which are associated with toxicity [26,27]. PD-L1 expression, quantified as the Tumour Proportion Score (TPS), is the only approved biomarker used in daily practice for the stratification of ICI therapies for NSCLC [3,22]. However, its relevance in *EGFRm* tumours is less straightforward [23,28]. Notably, studies have shown that increased PD-L1 expression in *EGFRm* NSCLC predicts worse outcomes with first-line TKIs but may improve the outcome with ICI therapy in later lines of treatment [29,30,31,32]. TKIs can also modulate the immune landscape of cancer, increasing the therapeutic potential of ICIs by boosting the cytotoxic CD8+ T cell population, increasing dendritic cell counts, and depleting Foxp3+ Tregs [33]. However, these beneficial changes are temporary and decrease with continued TKI treatment, counteracting the therapeutic benefit [33]. Therefore, combining EGFR-TKIs with ICIs can enhance the response of a subpopulation of NSCLC patients with *EGFRm* tumours resistant to first-line TKIs [4,34] despite the risk of additional adverse effects [26,35]. 

Recent studies highlighted the critical role of the tumour microenvironment (TME) in modulating the response to ICIs, particularly in *EGFRm* NSCLC [36,37]. These tumours frequently exhibit poor infiltration of inflammatory cells and a low level of immunogenicity, characterised by low PD-L1 expression, reduced tumour mutational burden, a low level of infiltration of CD8+ tumour-infiltrating lymphocytes, and activation of the immunosuppressive CD73/adenosine axis [38,39]. CD73, an ecto-5′-nucleotidase, works in concert with CD39 to regulate the immune response [40]. CD39, primarily expressed by regulatory T cells (Tregs), dendritic cells, macrophages, and endothelial cells, catalyses the conversion of ATP to AMP, a key step in the adenosine pathway. CD73, expressed by tumour cells, endothelial cells, fibroblasts, and immune T lymphocytes and Tregs, converts AMP to adenosine [36,37]. The resulting extracellular adenosine inhibits the immune cell function and promotes the formation of Tregs and myeloid-derived suppressor cells (MDSCs), enhancing their suppressive capabilities. This mechanism allows tumours to evade immune surveillance by inhibiting the anti-tumour functions of T cells and inducing T cell apoptosis [33,41,42]. 

Beyond its enzymatic role, the intracellular signalling pathway of CD73 has an impact on various events within cancer cells, including epithelial–mesenchymal transition (EMT) [43,44,45], cell cycle progression [46,47], and chemotherapy resistance via the Src and AKT pathway [48,49]. The co-expression of CD39 and CD73 on immune and tumour cells creates a highly immunosuppressive environment, facilitating tumour progression and resistance to therapies [50,51,52,53,54]. Given the elevated expression of CD73 in many LUAD cases [55] and its role in immune suppression, we aimed to investigate its expression using IHC in the context of *EGFRm* LUAD. Few studies have investigated CD73 expression in NSCLC using IHC, and only two have specifically assessed homogeneous cohorts of *EGFRm* LUAD [52,56]. Our study provides an original contribution to the literature by analysing a larger cohort of patients that are exclusively *EGFRm*- and treatment-naive. Findings across previous studies have been generally inconsistent [34,53,54,55,56,57,58,59,60], likely due to differences in cohort composition, sample types, histological and molecular subtypes, CD73 antibody used clones, and variability in IHC assessment and scoring methods. A detailed comparison of these studies is provided in Table 1. We conducted our study within the context of the development of anti-CD73 therapies as a potential approach to enhance TKI efficacy and overcome the resistance of *EGFRm* tumours. Combining anti-CD73 therapy, with or without anti-PD-L1 therapy, together with an EGFR-TKI may offer a promising strategy to improve the outcomes of patients with resistant *EGFRm* LUAD while reducing the associated toxicity [61,62]. As the prognostic value of expression of CD73 in NSCLC remains debatable [55], there is an urgent need to standardise methodologies for CD73 IHC evaluation by using larger, well-characterised patient cohorts to clarify the potential of CD73 as a predictive or prognostic marker, which could provide valuable insights into its suitability in guiding anti-CD73 therapeutic strategies. 

Our objective was to characterise the expression of CD73 in a unique cohort of 76 *EGFRm* LUAD patients who were treatment-naive and to correlate its expression with the level of PD-L1, clinicopathological and molecular features, as well as event-free survival (EFS).

## 2. Materials and Methods

### 2.1. Patients and Samples

Between May 2005 and June 2019, we retrospectively collected (Laboratory of Clinical and Experimental Pathology, Nice, France) data from 90 *EGFRm* LUAD patients encompassing all stages, which were obtained at diagnosis via bronchial or transthoracic biopsies, transbronchial needle aspiration (TBNA), and surgical resection. The study was conducted according to the Helsinki guidelines. Histological growth patterns in surgical resected samples were classified according to the WHO classification of tumours (5th edition) [64]. A high-grade pattern was identified in biopsy samples based on the presence of solid, micropapillary, or cribriform growth patterns. Clinicopathological data, including patient demographics, pathological tumour-node-metastasis (pTNM), stage (according to the 8th American Joint Committee on Cancer (AJCC) staging system and International Association for the Study of Lung Cancer (IASLC)) [65], PD-L1 expression, histological patterns, and molecular status, were available for all cases. 

Formalin-fixed paraffin-embedded (FFPE) tissues were processed for CD73 protein expression and *EGFR* amplification. Overall, 76 (76/90; 84%) cases were included for the IHC analysis, and 83 (83/90; 92%) cases were dedicated to the FISH analysis (Supplementary Figure S1).

### 2.2. Immunohistochemistry

IHC was performed on 3 μm thick formalin-fixed paraffin-embedded (FFPE) tissue sections placed on positively charged slides and using an automated staining system (BenchMark ULTRA; Ventana Medical Systems, Tucson, AZ, USA) with antibodies against CD73 (NT5E/CD73; clone D7F9A, dilution 1:150; Cell Signalling Technology) [57] and PD-L1 (clone 22C3, dilution 1:50; Dako Inc., Carpinteria, CA, USA) [66,67]. Expression of these proteins was detected using an OptiView Detection kit (Ventana) for PD-L1 and an Ultraview Detection kit (Ventana) for CD73 with a diaminobenzidine reaction to detect antibody labelling and haematoxylin counterstaining. PD-L1 expression was quantified routinely on tumour cells as the Tumour Proportion Score (TPS), as previously reported [68]. 

The expression level of CD73 was evaluated in tumour cells (CD73_TC_) in a semi-quantitative manner [53,57]. The percentage of positive tumour cells with any membrane staining (luminal, lateral, and complete), including the intensity of the staining (graded as follows: 0, non-staining; 1+, weak; 2+, median; or 3+, strong) was assessed. A minimum of 50 tumour cells were counted to ensure accuracy in the evaluation. The H-score was calculated to quantify the expression level of CD73 using the following formula: H-score = (% of cells at 1 + intensity × 1) + (% of cells at 2 + intensity × 2) + (% of cells at 3 + intensity × 3). This score ranges from 0 to 300, where a high score indicates high expression. A mean cut-off value of 150 was applied to categorise expression levels, with scores ≥ 150 considered as high and those < 150 considered as low. 

The Tumour Proportion Score (TPS) was calculated to determine the proportion of tumour cells expressing CD73. The TPS is defined as the percentage of viable tumour cells showing partial or complete membrane staining at any intensity and is calculated as follows: TPS = (Number of CD73 positive tumour cells/Number of total viable tumour cells) × 100. Expression levels with TPS values above 50% were classified as high, while those at or below 50% were considered low. Expression in stromal cells (CD73_SC_) was recorded as present or absent for lymphocytes, cancer-associated fibroblasts, and macrophages.

### 2.3. EGFR In Situ Fluorescent Hybridisation

*EGFR* FISH analysis was carried out using the LSI EGFR SpectrumOrange/CEP 7 SpectrumGreen probe (Vysis, Abbott Molecular, IL, USA) that recognised the *EGFR* gene and centromere 7. Tissue sections, 3 μm thick, were prepared for FISH staining; the process was performed according to the manufacturer’s instructions (with overnight hybridisation at 37 °C). For analyses, at least 60 nuclei were scored for signals using a Nikon Eclipse 80i microscope equipped with a triple-pass filter (DAPI/Green/Orange, Vysis) at a final magnification of 600×. Difficult cases were also analysed with the digital scanner (PathScan^®^ FISH, Excilone, Elancourt, France). The *EGFR* gene status was classified into six categories according to Capuzzo et al. [69,70]. Amplification was defined as one of the following aspects: (1) ≥15 copies of *EGFR* per cell in ≥10% of cells; (2) a ratio of the *EGFR* gene to chromosome 7 of ≥2; or (3) high polysomy ≥ 4 copies of *EGFR* per cell in ≥40% of analysed cell. 

### 2.4. EGFR Mutation Analysis

In this retrospective study, molecular analysis of the *EGFR* gene was performed using one of three ISO 15189-certified methods at the LPCE, as previously described: (1) pyrosequencing using the therascreen *EGFR* Pyro Kit (Qiagen, Hilden, Germany); (2) *EGFR* mutation assay on the Idylla platform (Biocartis, Mechelen, Belgium); or (3) next-generation sequencing with the Ion PGM NGS platform (Thermo Fisher Scientific, Waltham, MA, USA) [71,72,73]. To ensure consistency and comparability of the results, we focused exclusively on mutations that were detectable by all three methods.

### 2.5. Statistical Design and Analysis 

Categorical variables are expressed as n (%) and compared with the Chi-squared test or its non-parametric alternative Fisher’s test with simulated *p*-values. Numerical variables are expressed as the mean (standard deviation) or median [Interquartile Range] and compared to Student’s *t*-test or its non-parametric alternative Wilcoxon’s rank-sum test where appropriate. *p*-values are not adjusted. For survival analysis, Event-free survival (EFS) was defined as the time from diagnosis to an event that may include disease progression, recurrence of the disease, or cancer-related death. The Kaplan–Meier method with the log-rank test was used to compare survival curves in two different groups. *p*-values < 0.05 were considered significant. Statistical tests and representations of the data were performed using GraphPad PRISM (version 10.1.2) and STATAID software (https://github.com/VincentAlcazer/StatAid, accessed date 1 September 2023) [74]. 

## 3. Results

### 3.1. Patients and Characteristics of Samples

The clinical and pathological characteristics of the 76 LUAD patients are shown in Table 2. 

### 3.2. Analysis of Expression of CD73 and Correlation with Clinicopathological Features

#### 3.2.1. CD73 in Adjacent Non-Tumour Lung Tissue

**Figure 1:**
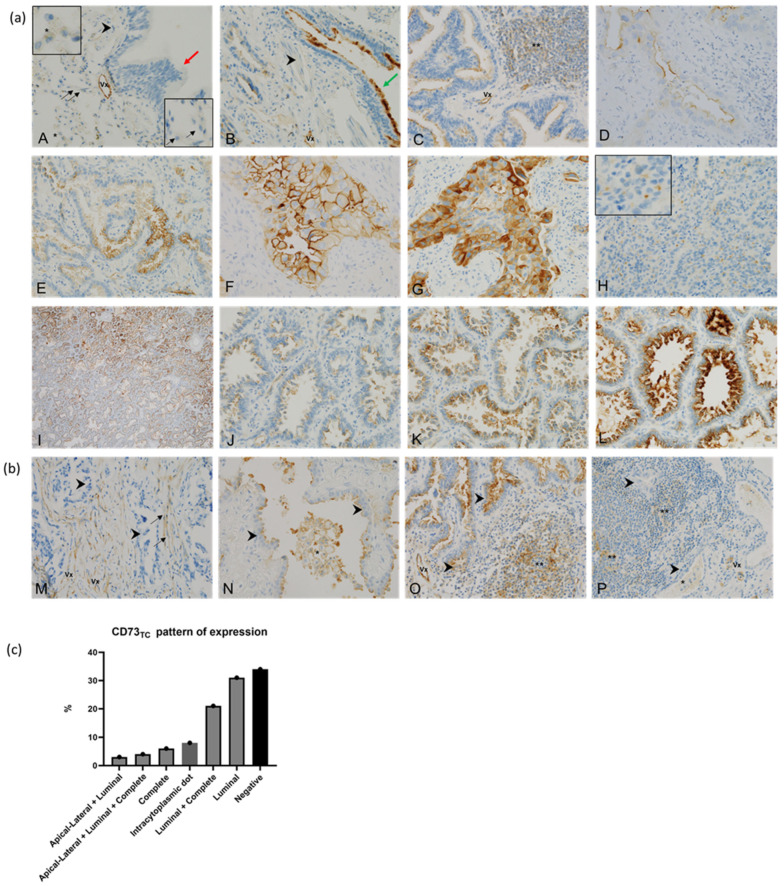
Expression of CD73. (**a**) Representative image of expression of CD73 in adjacent normal lung tissue and tumour cells. Analysis of adjacent normal lung tissue showed consistent negative staining for CD73 in pneumocytes (black arrows and lower right insert), macrophages (black asterisk and upper left insert), and smooth muscle (black arrowheads) (**A**,**B**, ×40). Normal bronchial epithelium was mostly negative (red arrows) (**A**) but occasionally showed positive staining in apical ciliated cells (green arrow) (**B**). Blood vessels (Vx) were positive and served as internal control (**A**,**B**, ×40). In LUAD, CD73 was expressed on cancer cell membranes with luminal (**C**,**D**, ×40), apico-lateral (**E**, ×40), or complete membrane staining (**F**,**G**, ×40) with infrequent cytoplasmic reinforcement (**G**, ×40). Intracytoplasmic dot staining in tumour cells (**H**, ×40) and positive tumour-infiltrating lymphocytes ** (**C**, ×40) were also observed. Intensity of CD73 staining varied among LUAD, and within same adenocarcinoma (**I**, ×4), with weak (1+) (**J**, ×40), medium (2+) (**K**, ×40), and strong (3+) (**L**, ×40) intensity staining, highlighting complexity of calculated H-score. (**b**) Representative image of expression of CD73 in stromal cells. Cancer-associated fibroblasts (black arrow) exhibited CD73 overexpression in 3% of cases, even when LUAD cells (black arrowheads) were negative (**M**, ×40). Stromal fibroblasts can be difficult to differentiate from small vessels (VX) in neovascularised tumours (**M**, ×40). In addition, CD73 expression was observed in macrophages (*) and tumour-infiltrating lymphocytes (**) in subset of cases (**N**,**O**,**P**, ×40) regardless of expression of CD73 in cancer cells. (**c**) Bar graph illustrating different patterns of expression of CD73 in tumour cells. Patterns observed include complete membrane staining, apical–lateral staining, luminal staining, and intracytoplasmic dot staining. These expression patterns often coexisted within same tumour sample.

In normal adjacent lung tissue, staining for CD73 was not observed in pneumocytes, smooth muscle, or intra alveolar macrophages. However, positive staining for CD73 was observed in the apical part of ciliated epithelial cells of the respiratory bronchial epithelium and in the endothelial cells of blood vessels (Figure 1a).

#### 3.2.2. CD73 in Tumour Cells (CD73_TC_)

The tumour cells exhibited four patterns of expression of CD73: (i) complete membrane staining, (ii) apical–lateral staining, (iii) luminal staining (in glandular structures), and (iv) intracytoplasmic dot staining (Figure 1a). These patterns coexisted within the same tumour sample, observed in 28 out of 76 cases (37%) (Figure 1c). Among the evaluated cases, 50/76 (66%) exhibited positive membranous expression of CD73, with 24/50 (48%) showing complete membrane staining. The TPS ranged from 5% to 100% with a median of 10%, while the H-score varied between 0 and 280 with a median of 20. A high expression of CD73_TC_ was detected in 12/76 samples (16%) based on the TPS and 11/76 samples (14%) based on the H-score. Both scoring methods yielded comparable results, with only one patient displaying discrepant values (TPS = 80%, H-score = 120) (Supplementary Figure S2). Given this consistency, all subsequent results are reported using the TPS.

No statistically significant correlations were found between the pattern of CD73_TC_ expression and clinicopathological parameters. CD73_TC_ expression, quantified using the TPS, showed no significant association with histological subtype, grade, vascular invasion, mitotic index, necrosis, TTF-1 expression, stage, smoking history, age, or sex. Similarly, no significant difference in CD73 expression was observed between primary tumours and metastatic sites. Additionally, no correlation was identified between CD73_TC_ expression and molecular biomarkers, including sensitive or resistant *EGFR* mutations and *EGFR* amplification. However, tumours with high TPS CD73_TC_ expression were significantly more likely to exhibit a complete membranous staining pattern (9/12, 75%; *p* = 0.015) (Table 3).

**Table 3:** Descriptive clinicopathological data related to TPS CD73_TC_ expression level.

Variable (n = 76, Unless Stated Otherwise)	Type	HIGH CD73_TC_ (TPS > 50%)	LOW CD73_TC_ (TPS ≤ 5 0%)	*p*-Value
Clinical and follow-up data				
Age at diagnosis	Mean (sd)	66 (7)	66.48 (11.07)	0.9525
	Median [IQR]	67 [60–71]	67 [63–74]	
Sexe	Female	9 (75)	43 (67.19)	0.8447
	Male	3 (25)	21 (32.81)	
Smoking (n = 72)	Smoker	5 (41.67)	24 (40)	1
	Non-smoker	7 (58.33)	36 (60)	
Stage category	Advanced	5 (42)	37 (58)	0.4740
	Early	7 (58)	27 (42)	
Brain_metastasis (n = 53)	No	6 (75)	32 (71)	1.0000
	Yes	2 (25)	13 (29)	
OS_Month				
	Mean (sd)	75 (50)	41 (36)	0.0868
	Median [IQR]	65 [29–79]	28 [14–60]	
Pathological data				
Origin of samples	Metastasis	3 (25)	20 (31)	0.9282
	Primary	9 (75)	44 (695)	
Histology subtype (n = 39)	Acinar	2 (22)	13 (43)	0.35
	Cribriform	1 (11)	0 (0)	
	Lepidic	0 (0)	1 (3)	
	Papillary	5 (56)	12 (40)	
	Solid	0 (0)	2 (7)	
	Micropapillary	1 (11)	2 (7)	
High-grade component (n = 71)	No	6 (50)	36 (61)	0.6998
	Yes	6 (50)	23 (39)	
Emboli (n = 46)	No	3 (27)	14 (29)	1.0000
	Yes	8 (73)	34 (71)	
Immunohistochemistry data				
PD-L1 expression category (n = 73)	High	0 (0)	10 (17)	0.2467
	Moderate	2 (17)	5 (8)	
	Negative	10 (83)	46 (75)	
PDL1 % TC	Mean (sd)	1 (3)	13 (30)	0.44
	Median [IQR]	0 [0–0]	0 [0–0]	
CD73_TC_ pattern (n = 50)	Apical lateral	1 (8)	0 (0)	0.0152
	Complete	9 (75)	16 (42)	
	Luminal	2 (17)	22 (58)	
CD73_TC_ dot staining (n = 50)	Yes	3 (25)	5 (8)	0.11
	No	9 (75)	59 (92)	
CD73_SC_ in lymphocyte	Negative	12 (100)	57 (89)	0.5103
	Positive	0 (0)	7 (11)	
CD73_SC_ in macrophage	Negative	11 (92)	60 (94)	1.0000
	Positive	1 (8)	4 (6)	
Molecular data				
*EGFR* amplification status (n = 71)	Yes	9 (75)	38 (64)	0.7096
	No	3 (25)	21 (36)	
*EGFR* mutation at baseline	Ex18_G719	1 (8)	2 (3)	0.7366
	Ex20_S768I	0 (0)	2 (3)	
	L858R	5 (42)	23 (36)	
	del19	6 (50)	37 (58)	
*EGFR* mutation at baseline classification (Common vs. Uncommon)	Common	11 (92)	60 (94)	1.0000
	Uncommon	1 (8)	4 (6)	
T790M during follow-up (n = 21)	No	2 (100)	9 (47)	0.5007
	Yes	0 (0)	10 (53)	

**Figure 2:**
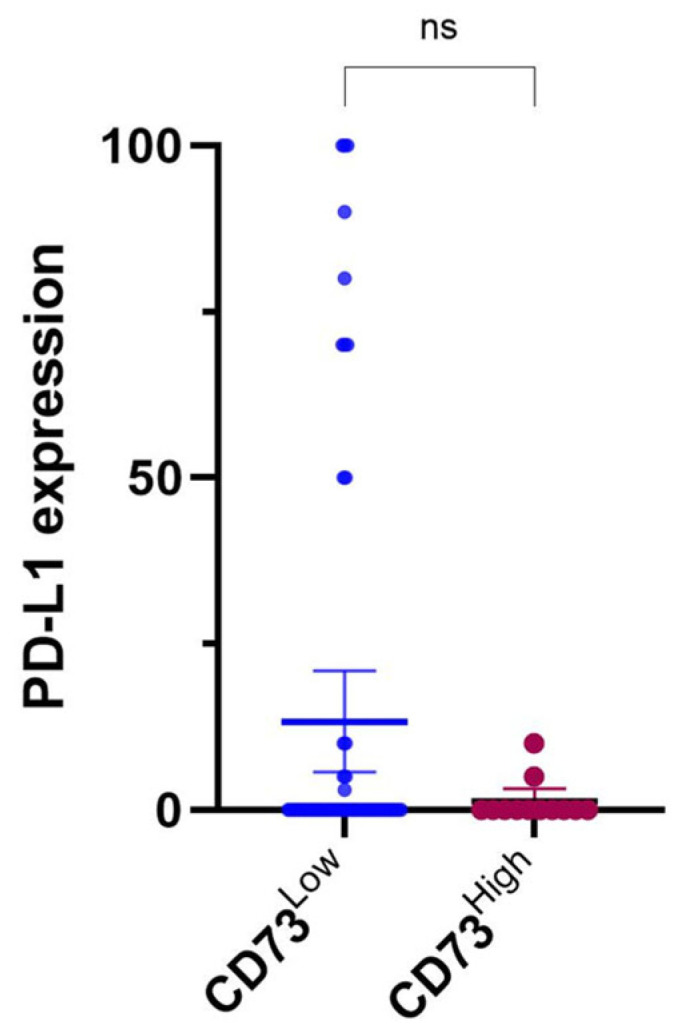
Comparison of PD-L1 expression and CD73 expression in tumour cells (CD73_TC_) using Tumour Proportion Score (TPS). Dot Tumour plot illustrating relationship between expression of PD-L1 and CD73 in tumour cells. High expression of both PD-L1 and CD73_TC_ was mutually exclusive. Plot suggests trend of inverse correlation, where high levels of PD-L1 are generally associated with low levels of expression of CD73. However, this correlation is not statistically significant.

CD73_TC_ and PD-L1 were co-expressed in 11/73 (15%) of cases. Among PD-L1-negative cases, 62% (37/60) were positive for CD73_TC_. The high expression of both PD-L1 and CD73_TC_ was mutually exclusive. Notably, a higher proportion of tumours in the high CD73 category were PD-L1-negative (83%; 10/12), although this trend did not reach statistical significance (*p* = 0.44) (Figure 2). To further investigate this relationship, we performed Pearson correlation analyses, which revealed a weak inverse correlation between CD73 and PD-L1 expression (CD73 H-score: r = −0.1639, *p* = 0.1657; CD73 TPS: r = −0.2, *p* = 0.1646), though this was not statistically significant (Supplementary Figure S3).

The scatter plots illustrate this distribution and suggest a potential inverse trend. However, this association remains inconclusive due to high inter-sample variability and a lack of statistical significance.

#### 3.2.3. CD73 in Stromal Cells (CD73 SC)

CD73_SC_ was expressed in cancer-associated fibroblasts in 2/76 cases (3%), while macrophages or infiltrating lymphocytes were positive in 12/76 (16%) of cases (Figure 1b). The expression of CD73_SC_ was more frequently observed in an early tumour stage (*p* = 0.037), PD-L1-negative tumours (*p* = 0.030), non-amplified *EGFR* tumours (*p* = 0.0018), and *EGFR* L858R-mutated tumours (*p* = 0.067) (Table 4).

### 3.3. EGFR Amplification and Correlation with Clinicopathological Features and Expression of CD73

The amplification of *EGFR* was observed in 70% (58/83) of cases (Supplementary Figure S4). Testing was uninformative for FISH in 8% (7/90) of the samples due to an inadequate amount of material or lack of hybridisation. Given that amplified tumours exhibit heterogeneous clinical features, we analysed their association with key clinicopathological parameters. No significant correlations were found between *EGFR*-amp and stage, smoking history, PD-L1, and *EGFR* mutation type. However, *EGFR*-amp was significantly associated with a high-grade pattern (*p* = 0.016) (Supplementary Table S1). 

### 3.4. Event-Free Survival and Follow-Up 

**Figure 3:**
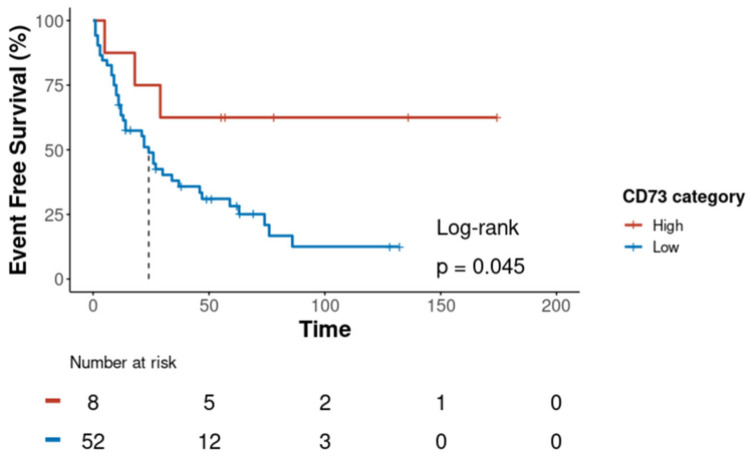
Kaplan–Meier curves illustrating event-free survival in patients categorised by high and low levels of CD73 expression in tumour cells (CD73_TC_) using the Tumour Proportion Score (TPS). Patients with a high level of expression of CD73_TC_ (red line) had a significantly extended event-free survival in contrast to patients with a low level of expression of CD73_TC_ (blue line). The median event-free survival for patients with a low level of expression of CD73_TC_ was 24 months, while for those with a high level of expression of CD73, it was yet to be reached (log-rank test, *p* = 0.045 with TPS). The number of patients at risk at each time point is specified below the *x*-axis.

Shorter event-free survival (EFS) was significantly associated with being male (*p* = 0.019), advanced stage (*p* < 0.001), high-grade component (*p* = 0.016), and high PD-L1 (*p* = 0.025) (Table 5). High CD73_TC_ expression was correlated with longer EFS in the univariate analysis, as shown by the median survival not being reached after 34 months of follow-up (*p* = 0.045) (Figure 3). However, in the multivariate analysis, which included CD73 expression, PD-L1 expression, stage, and age, only disease stage remained statistically significant (*p* = 0.0078) (Supplementary Figure S5). To further explore the impact of tumour stage, we performed an exploratory subgroup analysis, stratifying patients into early-stage (Stages I–IIIA) and advanced-stage (Stages IIIB–IV) disease (Supplementary Figure S6). In early-stage patients, a trend towards improved EFS in the high CD73_TC_ expression group was observed, but this did not reach statistical significance (*p* = 0.11). In advanced-stage patients, no significant association was observed between CD73 expression and EFS (*p* = 0.91).

*EGFR*-amp demonstrated no significant association with EFS. In patients treated with EGFR TKIs, follow-up was available in 32/44 (73%) patients with a median of 29 months (range 17–54.5), showing no statistical correlation between EFS and the expression of CD73_TC_ (*p* = 0.92) (Supplementary Table S2; Figure S7).

## 4. Discussion

Recent evidence highlights the emerging role of CD73 inhibitors in treatment decision-making for LUAD [34,63]. Preclinical studies have demonstrated the therapeutic potential of targeting the CD73/adenosine pathway in *EGFRm* lung cancer. In an immunocompetent mouse model, CD73 was found to be upregulated, and its blockade significantly inhibited tumour growth [36,37]. Furthermore, in an independent xenograft mouse model, the combination of anti-PD-L1 and anti-CD73 therapy enhanced T cell-mediated killing of *EGFRm* NSCLC, whereas monotherapy with either antibody was ineffective [54]. These findings suggest that the dual inhibition of CD73 and PD-L1 may enhance immune responses and could be a promising therapeutic strategy for LUAD.

In human *EGFRm* tumours, targeting CD73 shows promising results, as demonstrated by ongoing clinical trials testing adenosine pathway inhibitors, either as monotherapy or in combination with ICIs and/or TKIs [56,61,63,75,76]. CD73 inhibition may sensitise tumours to treatment by reducing adenosine production, thereby enhancing T cell and dendritic cell activity while suppressing the immunosuppressive functions of regulatory T cells and [63]. Additionally, the inhibition of CD73 improves the efficacy of anti-PD-1 and anti-CTLA-4 monoclonal antibodies [50], inhibits tumour growth, and reverses the exhausted T cell phenotype in mouse models [77]. 

Among CD73-targeting agents, the human monoclonal antibody Oleclumab (MEDI9447) exhibits high specificity towards CD73, inhibiting its enzymatic activity by sterically blocking and cross-linking CD73 dimers. Additionally, it reduces CD73 expression through internalisation, preventing the production of immunosuppressive extracellular adenosine [51].

While CD73 inhibition may enhance ICI efficacy, it could also exacerbate inflammatory side effects, particularly in *EGFRm* patients, who are known to be at a higher risk of immune-related toxicities when treated with ICIs. Clinical data on anti-CD73 therapy in *EGFRm* patients remain limited; however, early findings suggest a favourable safety profile with a low incidence of severe adverse effects and a low rate of treatment discontinuation [34,56]. Furthermore, no additional toxicities have been reported beyond those already known with durvalumab monotherapy [63]. 

In this context, we explored the feasibility of using IHC to establish a correlation between the expression of CD73 and clinicopathological data, thus identifying a potential predictive biomarker. We performed two methods for the quantitative evaluation of CD73_TC_. Both the TPS and H-score produced similar results within the context of *EGFRm* LUAD. Statistical analyses underscored the consistency of these two scoring systems when considering the clinicopathological data. Yet, the increased complexity of the H-score does not augment precision within our limited patient cohort and may hamper reproducibility among pathologists. We tested the cut-offs published in the literature but did not observe significant results in our cohort. Therefore, we opted for a mean cut-off based on the average score of the total range: 150 out of 300 for the H-score and 50 out of 100 for the TPS.

Consistent with the existing literature, we demonstrate the absence of expression of CD73 in normal alveolar tissue and expression in tumour cells, reflecting 66% CD73_TC_ positivity, in line with prior data linking the expression of CD73 to *EGFR* mutations [54,57,58]. Endothelial cells and the respiratory bronchial epithelium could serve as internal controls. This is further supported by the literature, which documents similar observations in the function of endothelial cells [78] and in human oviducts, where CD73 regulates ciliary beat frequency and mucociliary clearance [79]. We also report for the first-time intracytoplasmic dot staining of CD73_TC_ in lung cancer tissue; however, we did not observe any correlation between this staining pattern and clinicopathological parameters. Interestingly, Alcedo et al. previously documented para-nuclear expression in hepatocellular carcinoma cells, with co-localisation observed at the Golgi complex. This discovery highlights the abnormal glycosylation of CD73 in tumour hepatocytes, leading to the mislocalisation and functional suppression of CD73. This insight suggests a potential new therapeutic target for improving the treatment outcome by addressing this specific regulatory mechanism [80]. 

While the role of CD73 in tumour progression has been well documented [42,81,82,83,84], our investigations into the correlation between CD73_TC_ and tumour emboli, metastasis, high grade, and stage did not yield any significant associations.

Regarding immune checkpoints, the co-expression of CD73_TC_ and PD-L1 was rare, occurring in only 15% of cases. Moreover, the high expression of both PD-L1 and CD73_TC_ was mutually exclusive, with a trend towards an inverse association between the high expression of CD73_TC_ and reduced PD-L1 expression in tumour cells. However, a correlation analysis between CD73 and PD-L1 expression did not reach statistical significance (r = −0.2, *p* = 0.16), indicating that while an inverse trend may exist, these markers are largely independent in our cohort. Previous studies [53,57,58] identified a correlation between high CD73 and increased PD-L1 expression, either in smaller cohorts of 15 to 25 *EGFR* tumours or in cohorts analysed after TKI treatment [52] (Table 1). Unlike these studies, our research focused on a more homogeneous and larger cohort, providing insights into a broader patient population, albeit under different treatment conditions. These results suggest the presence of distinct immune evasion mechanisms in *EGFRm* LUAD. The inverse relationship could indicate that tumours with a high expression of CD73 may rely on the adenosine pathway for immune suppression\ rather than on PD-L1-mediated immune checkpoint inhibition. Moreover, these findings highlight the complexity of the tumour immune microenvironment, suggesting that tumours may employ diverse strategies to escape immune surveillance. This variability could have significant implications for the development of targeted therapies, highlighting the need for personalised approaches that address these distinct immune evasion mechanisms. It could be of strong interest to further investigate CD73’s interactions with immune cells. However, given the limitations of our cohort—notably the low proportion of TILs+ cases and limited CD73 stromal expression—we prioritised a tumour-centred approach.

Of interest, we observed a correlation between a high CD73_TC_ level and improved EFS at baseline, suggesting potential prognostic significance. To further investigate this association, we performed a multivariate Cox proportional hazards model incorporating CD73_TC_ expression, PD-L1 expression, disease stage, and age. While a univariate analysis identified a significant correlation between high CD73_TC_ expression and improved EFS, this association was no longer significant in the multivariate model, likely due to the limited sample size (60 patients with available EFS data). In this adjusted model, only the disease stage remained statistically significant, confirming its well-established prognostic impact. CD73 expression was not correlated with tumour stage (Table 3), indicating that the observed association with EFS is not solely driven by stage distribution. These findings suggest that while CD73 may play a role in tumour progression, larger studies are needed to fully elucidate its independent prognostic value in *EGFRm* LUAD. This association has previously been reported only once by Ishii et al., who demonstrated that in a cohort of 25 treated *EGFRm* LUAD patients, a high expression of CD73_TC_ was linked to improved progression-free survival and overall survival [53]. However, the prognostic implications of the expression of CD73 have been inconsistently reported, with some studies showing no correlation [34,57,58] and others suggesting an association with worse prognosis [55,56,60] (Table 1). Several factors may explain these discrepancies. One key distinction is that our cohort includes a higher proportion of early-stage tumours (43%), whereas most previous studies focused on advanced-stage disease. Our exploratory subgroup analysis (Supplementary Figure S6) indicated that the association between high CD73_TC_ expression and improved EFS is more apparent in early-stage tumours, though it does not reach statistical significance. These findings suggest that CD73 may have a more prominent role in early tumour progression, where immune evasion mechanisms could differ from those in advanced disease. However, the absence of statistical significance in early-stage tumours and the limited sample size of our cohort warrant cautious interpretation. Further validation in larger independent cohorts is necessary to determine whether CD73 has stage-dependent prognostic implications and to clarify its potential role in tumour progression. 

In contrast to some studies reporting an association between CD73 expression and poorer survival in *EGFRm* patients treated with TKIs [56], we did not observe a significant correlation between baseline CD73_TC_ expression and survival in our TKI-treated subgroup. Differences in sample size, treatment heterogeneity, and patient selection criteria may explain these discrepancies. Additionally, Ishii et al. previously reported that high CD73_TC_ expression was linked to improved progression-free and overall survival in a small cohort of 25 treated *EGFRm* LUAD patients, highlighting the inconsistency in CD73’s prognostic impact across studies. Previous reports have suggested that TKI treatment may modulate the tumour immune microenvironment, potentially influencing CD73 expression patterns and their prognostic significance. Specifically, studies have indicated that CD73 expression may increase upon TKI resistance, particularly in tumours with acquired PD-L1 positivity [52]. However, our study focused exclusively on CD73 expression at baseline, before TKI exposure, allowing us to assess its potential prognostic role independently of treatment-induced changes. This distinction may explain why our findings differ from those observed in cohorts enriched with TKI-resistant patients.

Beyond cohort composition and treatment variability, technical factors may also contribute to the observed differences. Variations in IHC interpretation and scoring criteria could influence the reported correlations. Additionally, the inverse expression pattern between CD73 and PD-L1, a biomarker known to impact prognosis, may play a role. Another important limitation is the reduced follow-up duration for some patients, which may affect the assessment of long-term outcomes.

Although the analysis of the expression of CD73_SC_ is limited in our cohort, we observed a trend towards better prognosis, as shown in Table 4. The association of the expression of CD73_SC_ in early-stage tumours and PD-L1 negativity indicates that these tumours may have a less aggressive phenotype and a better response to therapy. Additionally, the lack of *EGFR* amplification and the presence of the common *EGFR* L858R mutation, both of which are linked to better responses to EGFR-TKIs, further support the idea that the expression of CD73_SC_ may be indicative of a more favourable clinical outcome. This is consistent with previous reports suggesting that CD73 in the stroma plays an anti-tumour role by reducing NF-κB signalling in tumour cells [85,86].

Despite the known influence of EGFR signalling on the expression of CD73, our investigation did not establish a direct relationship between *EGFR-amp* and the expression of CD73. While FISH is commonly used to detect an *EGFR* amplification, the results often reflect chromosome 7 polysomy rather than a true gene amplification. Amplification and polysomy, although distinct genetic events, both lead to EGFR protein overexpression, activating downstream signalling pathways that promote tumour growth and survival [87,88]. Consequently, they are often considered to be equivalent from a biological perspective. This complexity poses challenges in accurately classifying cases as amplification or high polysomy [89]. Consistent with previously published data [90,91], the proportion of *EGFR-amp* was high (70%, n = 58) when scored with the method described by Capuzzo et al. [69]. *EGFR-amp* is often associated with poor outcomes, invasion, and metastasis [91,92]. However, the literature presents conflicting findings [17,87,90,93,94,95,96], and a recent study by Chmielecki et al. concerning the FLAURA study found no significant association between baseline *EGFR-amp* and a suboptimal treatment response to first-line osimertinib [16]. Our study likewise did not establish a significant association between *EGFR-amp*, disease stage, and EFS. 

While IHC remains a widely used technique to assess the expression of CD73, its accuracy in reflecting the enzyme’s true biological activity is questionable. Protein expression, detected with IHC, does not always correlate with enzymatic activity. Emerging techniques to detect the activity of CD73 include liquid biopsy, which offers a less invasive and potentially more accurate assessment of the enzymatic function [97,98]. Exosomes are small extracellular vesicles that carry proteins, lipids, and nucleic acids, reflecting the molecular composition of their cell of origin. Studies have shown that soluble CD73 in serum and exosomal CD73 can be a reliable indicator of enzymatic activity, providing a real-time snapshot of the tumour biology. These biomarkers may serve as indicators for disease progression and response to therapy, as demonstrated in melanoma [99] and prostate cancer [100], suggesting a similar potential in lung cancer.

Ongoing research aimed at characterising the unique features of tumour biology and the TME in *EGFRm* NSCLC, as well as identifying patient subgroups with enhanced responses to ICI therapy, is promising. Considering the prevalence of *EGFR* mutations and amplifications in LUAD and their association with restricted responses to ICIs, it is imperative to conduct large-scale prospective studies to comprehensively investigate the expression of CD73 as a potential prognostic factor and predictor of the response to immunotherapy and establish standardised methodologies to assess its expression. Moreover, future research should focus on validating liquid biopsy techniques in clinical settings and on exploring their utility in conjunction with traditional IHC. 

## 5. Conclusions

The present study is the first comprehensive investigation to explore the expression of CD73 in LUAD with different *EGFR* genomic alterations. The observed upregulation of expression of CD73_TC_ at baseline, along with its association with a longer EFS and lower expression of PD-L1, highlights its potential importance in the evolving landscape of immunotherapy and targeted treatments for *EGFR*-mutated NSCLC. This suggests the presence of a distinct immune TME profile in *EGFRm*-LUAD, characterised by distinct mechanisms of immune evasion driven by specific molecular pathways. Understanding these unique profiles could have significant therapeutic implications, particularly in the context of combined therapies, including CD73 blockers and ADORA receptor inhibitors to maximise CD73 blockade, along with conventional ICIs.

Such insights may lead to the development of more effective treatment strategies, enhancing the efficacy of immunotherapies by targeting the specific pathways involved in immune resistance within this subset of lung cancer. 

## Figures and Tables

**Table 1:** Comprehensive review of CD73 immunohistochemical expression: correlations with clinicopathological features and treatment outcomes.

											CD73_TC_ Expression and Correlation with						
References	Type of Study	No. of Cases	Sample	Stage	Histological Subtype	No. of *EGFR* Mutant	Treatment History	IHC (Clone and Dilution)	Interpretation	Cut-Off	Histological Pattern	Patients Characteristics	*EGFRm*	TMB	PDL1	Survival	Response to ICI
Inoue, 2017 [55]	retrospective	642	FFPE (TMA)	I-III	SCC; LUAD; others	119 (protein expression)	before or after chemo	D7F9A 1:200	H-score	low < 162 < high	Yes (LUAD; TTF-1; ALK)	Yes (female; never smoked)	Yes (based on EGFR protein expression)	_	_	Yes (whole cohort; shorter OS and RFS)	_
Isomoto, 2020 [52]	retrospective	70	FFPE	Advanced	LUAD	70	before and after TKI	D7F9A	H-score TILs		_	_	_	_	Yes (High PD-L1, after TKI)	_	_
Giatromanolaki, 2020 [58]	retrospective	98	FFPE	I-III	SCC; LUAD; others	_	treatment-naive	EPR6114 1:200	TPS CAF (% stained stroma area)	5% < low < 40% 50% < medium < 70% 80% < high < 100%	Yes Inverse correlation between CD73_TC_ and CAF Less CD73_SC_ expression in stage I	No	_	_	No	No	_
Ishii, 2020 [53]	retrospective	91	FFPE	Advanced	NSCLC	25	after TKI	D7F9A	TPS	≥50%	_	No	No	_	Yes (high PD-L1)	Yes (longer PFS, OS)	Yes (better ORR in *EGFRm*)
Ramdani, 2021 [59]	retrospective	48	FFPE	Advanced	SCC; LUAD; others	3	treatment-naive	1D7	no or heavy staining for TC and IC	_	_	_	_	_	_	No	No
Rocha, 2021 [57]	retrospective	106	FFPE (TMA)	I–III	LUAD	15	treatment-naive	D7F9A 1:200	TPS luminal; basolateral and total stain	T negative ≤ 1%; 1% > T low < 55%; T high ≥ 55%	Yes (solid)	Yes (smoking; sex)	No	Yes	Yes (high PD-L1)	No	_
Tu, 2022 [54]	retrospective	231	FFPE	_	NSCLC	99	_	EPR6115	TPS luminal or complete stain	_	_	_	Yes	_	_	_	_
Herbst, 2022 [34]	prospective	107	FFPE	III	NSCLC	_	treatment-naive	D7F9A 1:200	TPS	low < 10 ≤ high	_	_	_	_	_	No	No
Bendell, 2023 [63]	retrospective	126	FFPE	Advanced	LUAD; PDAC, CRC	42	after treatment	EPR6115	TPS	>10%	_	_	_	_	_	_	_
Kim, 2023 [56]	retrospective	26 paired tumour	FFPE	_	LUAD	26	before and after TKI 1st generation	D7F9A 1:100	H-score	low < 60 ≤ high	_	_	_	_	_	Yes (shorter PFS with 1st generation TKI)	_
Haratani, 2023 [60]	prospective	135	FFPE	III	NSCLC	13	treatment naive	D7F9A 1:200	H-score IC	low < 70 < high	Yes (NS-NSCLC)	_	No	_	_	shorter PFS (with ICI)	_

Abbreviation: CAF: Cancer-Associated Fibroblasts; Chemo: Chemotherapy; CRC: Colorectal Cancer; FFPE: Formalin-Fixed, Paraffin-Embedded; IC: Immune Cells; ICI: Immune Checkpoint Inhibitor; LUAD: Lung Adenocarcinoma; NSCLC: Non-Small Cell Lung Cancer; OS: Overall Survival; PDAC: Pancreatic Ductal Adenocarcinoma; PD-L1: Programmed Death-Ligand 1; PFS: Progression-Free Survival; RFS: Recurrence-Free Survival; SCC: Squamous Cell Carcinoma; TC: Tumour Cells; TILs: Tumour-Infiltrating Lymphocytes; TKI: Tyrosine Kinase Inhibitors; TMA: Tissue Microarray; TMB: Tumour Mutational Burden; TPS: Tumour Proportion Score. Symbol “-“: Not assessed.

**Table 2:** Characteristics of 76 enrolled patients with *EGFR*-mutated LUAD.

Variable (n = 76 Unless Stated Otherwise)	Type	Whole Cohort, n (%) Unless Stated Otherwise
Clinical and follow-up data		
Age at diagnosis	Median [IQR]	67 [62–73]
Sex	Female	52 (68)
	Male	24 (32)
Follow-up	Median [IQR]	29 [14–63]
Smoking status	Former smoker	10 (13)
	Smoker	19 (25)
	Non-smoker	43 (65)
Stage category	Advanced	42 (55)
	Early	34 (45)
Brain metastasis (n = 53)	Yes	15 (28)
	No	38 (72)
Treatment type (n = 59)	Including TKI	44 (75)
	Including immunotherapy	4 (7)
	Chemotherapy alone	11 (19)
	Radiotherapy alone	3 (5)
Number of treatments (n = 76)	Mean (sd)	1 (1)
	Median [IQR]	1 [0–2]
Status at end of follow-up (n = 49)	Dead	23 (47)
	Alive	26 (53)
Pathological data		
Type of sample	Biopsy	36 (47)
	Cytology	1 (2)
	Surgical specimen	39 (51)
Origin of samples	Metastasis	23 (30)
	Primitive	53 (70)
High-grade component (n = 71)	No	42 (60)
	Yes	29 (41)
Type of high-grade component (n = 23)	Cribriform	7 (31)
	Micropapillary	4 (17)
	Solid	12 (52)
Necrosis (n = 47)	No	32 (68)
	Yes	15 (32)
Emboli (n = 59)	No	17 (29)
	Yes	42 (71)
Mitosis (n = 45)	No	5 (11)
	Yes	40 (89)
Immunohistochemistry data		
TTF1 expression on tumour cells	Negative	1 (1)
	Positive	75 (99)
Percentage of PD-L1 expression on tumour cells (n = 73)	Mean (sd)	11 (28)
	Median [IQR]	0 [0–5]
PD-L1 expression category (n = 73)	Negative (<1%)	56 (77)
	Moderate (1–49%)	7 (9)
	High (≥50%)	10 (14)
CD73 tumour expression (n = 76)	Negative	26 (34)
	Positive	50 (66)
Type of CD73 staining (n = 50)	Apical–lateral	5 (7)
(co-existing within same tumour)	Complete	24 (48)
	Luminal	44 (88)
	Dot intracytoplasmic	8 (16)
Percentage of CD73 tumour staining (n = 76)	Mean (sd)	27 (31)
	Median [IQR]	10 [0-51]
Percentage of CD73 tumour expression 1+ (n = 76)	Mean (sd)	5 (10)
	Median [IQR]	0 [0–5]
Percentage of CD73 tumour expression 2+ (n = 76)	Mean (sd)	22 (28)
	Median [IQR]	10 [0–40]
Percentage of CD73 tumour expression 3+ (n = 76)	Mean (sd)	33 (59)
	Median [IQR]	0 [0–53]
CD73 H-score (n = 76)	High	11 (14)
	Low	65 (86)
CD73 TPS (n = 76)	High	12 (16)
	Low	64 (84)
CD73 expression on lymphocytes (n = 76)	Negative	69 (91)
	Positive	7 (9)
CD73 expression on macrophages (n = 76)	Negative	71 (93)
	Positive	5 (7)
CD73 expression on CAFs (n = 73)	Negative	71 (97)
	Positive	2 (3)
Molecular data		
*EGFR* mutation at baseline	Ex18_G719	3 (4)
	Ex20_S768I	2 (3)
	L858R	28 (37)
	del19	43 (56)
T790M during follow-up (n = 21)	No	11 (52)
	Yes	10 (48)

**Legends:** IQR: Interquartile Range; sd: Standard Deviation; TTF1: Thyroid Transcription Factor 1; PD-L1: Programmed Death-Ligand 1; TPS: Tumour Proportion Score; *EGFR*: Epidermal Growth Factor Receptor; Ex: Exon; del: Deletion; T790M: specific mutation in EGFR gene that confers resistance to certain TKIs; LUAD: Lung Adenocarcinoma; CAF: Cancer-Associated Fibroblast.

**Table 4:** Association between CD73 expression in stromal cells (recorded as present (positive) or absent (negative)) and clinic pathological data.

Variable (n = 76, Unless Stated Otherwise)	Type	CD73_SC_ Negative, n (%)	CD73_SC_ Positive, n (%)	*p*-Value
STAGE	Advanced	38 (61)	4 (29)	0.0372
	Early	24 (39)	10 (71)	
*EGFR* mutation at baseline	Ex18_G719	2 (3)	1 (7)	0.0670
	Ex20_S768I	2 (3)	0 (0)	
	L858R	19 (31)	9 (64)	
	Del19	39 (63)	4 (29)	
PD-L1 expression (n = 73)	Moderate/high	17 (29)	0 (0)	0.0303
	Negative	42 (71)	14 (100)	
PDL1 category	High	10 (16.95)	0 (0)	0.0710
	Moderate	7 (11.86)	0 (0)	
	Negative	42 (71.19)	14 (100)	
EGFR amplification (n = 71)	Amplified	44 (75)	3 (25)	0.0018
	Not amplified	15 (25)	9 (75)	

**Table 5:** Event-free survival analysis based on clinical and molecular variables.

Variable	No. of Patients with Data	Median Survival, Month	HR (95%CI)	*p*-Value
Sex			2.5 (1.4–4.4)	0.019
Male	51	12		
Female	22	30		
Stage			5.4 (3.0–9.9)	<0.001
Advanced	36	14		
Early	33	76		
High-grade component			2.6 (1.5–4.7)	0.016
Yes	29	18		
No	38	47		
PD-L1 expression			2.5 (1.2–4.9)	0.025
High	10	11		
Negative/moderate	58	27		
CD73 TPS			2.2 (1.0–4.9)	0.045
Low	52	24		
High	8	Not Reached		
CD73 H-score			2.0 (0.9–4.7)	0.092
Low	53	24		
High	7	Not Reached		

## Data Availability

The data presented in this study are available upon request from the corresponding author.

## Supplementary Materials

The following supporting information can be downloaded at https://www.mdpi.com/article/10.3390/cancers17061034/s1, Figure S1. Flowchart of sample selection and analysis; Figure S2. Dot plot illustrating correlation between expression of CD73 measured by TPS and H-score; Figure S3. Scatter dot plot illustrating correlation between expression of CD73 measured by TPS (and H-score); Figure S4. *EGFR* FISH assay; Figure S5. Multivariate Cox Regression Model adjusted for CD73_TC_ expression, PD-L1, stage, and age; Figure S6. Event-free survival (EFS) in early-stage (6a) and advanced-stage (6b) patients based on CD73_TC_ expression.; Figure S7. Kaplan–Meier curves illustrating event-free survival in patients treated with ITK categorised by high and low expression of CD73; Table S1. Comparison of clinicopathological features and molecular markers between patients with and without *EGFR* amplification; Table S2. Characteristics of patients treated with EGFR ITK.

## Abbreviations

The following abbreviations are used in this manuscript:

EFS	Event-Free Survival
EGFR	Epidermal Growth Factor Receptor
EGFR-amp	*EGFR-amp*
EGFRm	EGFR-Mutated
EMT	Epithelial–Mesenchymal Transition
FFPE	Formalin-Fixed Paraffin-Embedded
FISH	In Situ Fluorescent Hybridisation
IHC	Immunohistochemistry
LUAD	Lung Adenocarcinoma
MDSCs	Myeloid-Derived Suppressor Cells
NSCLC	Non-Small Cell Lung Cancer
SC	Stromal Cells
TC	Tumour Cells
TKIs	Tyrosine Kinase Inhibitors
TME	Tumour Microenvironment
TPS	Tumour Proportion Score
HIF1α	Hypoxia-Inducible Factor 1-alpha
LDH5	Lactate Dehydrogenase 5
