# Supplementary material for: Exploring the Expression of CD73 in Lung Adenocarcinoma with EGFR Genomic Alterations
Source: Cancers (Basel). 2025 Mar 20;17(6):1034. doi: 10.3390/cancers17061034 (PMC11941413; doi:10.3390/cancers17061034)

Figure S2. Dot plot illustrating the correlation between CD73 expression measured by TPS (and the H-score. Each point on the graph represents an individual patient.

Circle: TPS High (>50%) & H-score High (≥150); Cross: TPS Low (≤50%) & H-score Low (<150); Triangle: TPS High (>50%) & H-score Low (<150)

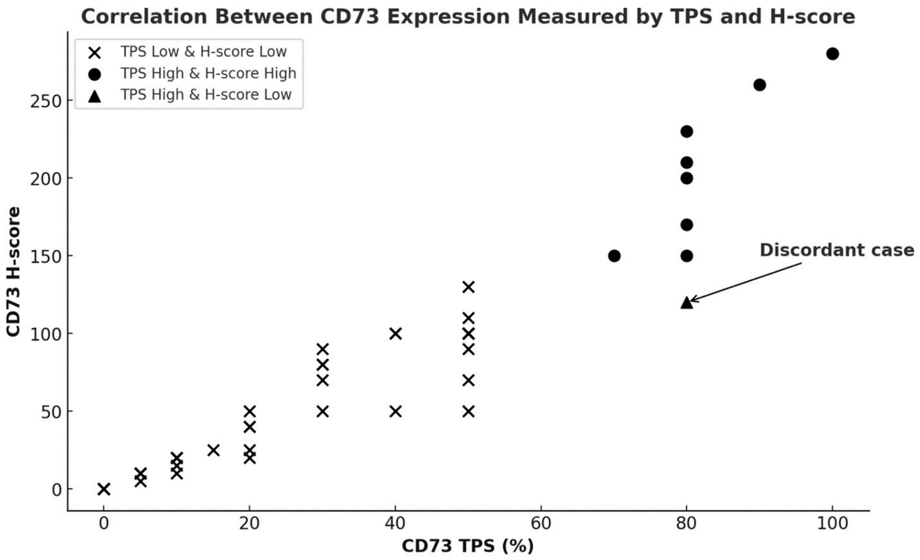

Supplement: Supplementary file 1 [file cancers-17-01034-s001.zip › Figure S2.pdf]
