# Supplementary material for: Exploring the Expression of CD73 in Lung Adenocarcinoma with EGFR Genomic Alterations
Source: Cancers (Basel). 2025 Mar 20;17(6):1034. doi: 10.3390/cancers17061034 (PMC11941413; doi:10.3390/cancers17061034)
Supplement: Supplementary file 1 [file cancers-17-01034-s001.zip › Table S1- EGFRamp and characteristics.pdf]

Table S1. Comparison of clinicopathological features and molecular markers between patients with *EGFR* amplification and those without. The values are displayed as mean (standard deviation) and median [Interquartile Range, IQR] for continuous variables, and as n (%) for categorical variables. The p-value indicates the statistical significance of the differences between the amplified and non-amplified groups.

**Variable n=83, unless stated**	**Type**	**Amplified**	**Not amplified**	**P value**
Age at diagnosis	Mean (sd)	68 (10)	68 (10)	0.882
	Median [IQR]	70 [63-74]	69 [65-74]	
Sex	Female	36 (62)	19 (76)	0.312
	Male	22 (38)	6 (24)	
Stage	Advanced	35 (60)	13 (52)	0.629
	Early	23 (40)	12 (48)	
High grade component (n=77)	No	24 (46)	19 (76)	**0.016**
	Yes	28 (54)	6 (24)	
Emboli (n=62)	No	8 (20)	7 (33)	0.347
	Yes	33 (80)	14 (67)	
PDL1 % (n=78)	Mean (sd)	17 (31)	6 (19)	0.084
	Median [IQR]	0 [0-10]	0 [0-0]	
PD-L1 category (n=78)	High	10 (19)	2 (8)	0.336
	Moderate	9 (17)	2 (8)	
	Negative	35 (65)	20 (83)	
$CD73_{TC}$ expression (n=71)	Negative	17 (36)	6 (25)	0.427
	Positive	30 (64)	18 (75)	
$CD73_{TC}$ Positive %	Mean (sd)	28 (31)	26 (29)	0.867
	Median [IQR]	10 [0-50]	18 [4-45]	
$CD73_{TC}$ TPS (n=71)	High (>50%)	9 (19)	3 (13)	0.739
	Low (≤50%)	38 (81)	21 (88)	
$CD73_{TC}$ H-score (n=71)	Mean (sd)	62 (78)	60 (72.12)	0.757
	Median [IQR]	20 [0-100]	35 [3.75-85]	
$CD73_{TC}$ H-score (n=71)	High (≥150)	9 (19)	2 (8)	0.312
	Low (<150)	38 (81)	22 (92)	
Event-free survival	Mean (sd)	46 (42)	45 (36)	0.861
	Median [IQR]	29 [15-61]	39 [16-62]	
EGFR mutation type	Ex18_G719	2 (3)	2 (8)	0.713
	Ex20_S768I	2 (3)	0 (0)	
	L858R	22 (37.93)	8 (32)	
	del19	32 (55.17)	15 (60)	
