# Supplementary material for: Exploring the Expression of CD73 in Lung Adenocarcinoma with EGFR Genomic Alterations
Source: Cancers (Basel). 2025 Mar 20;17(6):1034. doi: 10.3390/cancers17061034 (PMC11941413; doi:10.3390/cancers17061034)
Supplement: Supplementary file 1 [file cancers-17-01034-s001.zip › Table S2. Cohort treated with ITK clinicopathological data.pdf]

Supplementary Table S2. Characteristics of patients treated with ITK

Variable	Type	Whole cohort
**Clinical and follow-up data**		
Age at diagnostics	Mean (sd)	66.23 (10.97)
	Median [IQR]	68 [60.75-72.25]
Sexe	F	26 (59)
	M	18 (41)
Smoking	Smoker	12 (27)
	Former smoker	8 (19)
	Non smoker	23 (53)
stage	IA-1	1 (2)
	IB	3 (7)
	IIA	2 (4)
	IIB	2 (4)
	IIIA	4 (9)
	IIIB	3 (7)
	IIIC	1 (2)
	IV	28 (63)
Brain metastasis	no	20 (65)
	yes	11 (35)
Overall survival	Mean (sd)	39.47 (34)
	Median [IQR]	29 [17-54.5]
**Pathological data**		
Origin of samples	metastasis	14 (32)
	primitive	30 (68)
Type of samples	Biopsy	25 (57)
	Cytology	2 (5)
	Surgical resection	17 (39)
High grade component	no	18 (46)
	yes	21 (54)
Emboli	no	6 (18)
	yes	27 (81)
**Immunohistochemistry data**		
Percentage of PD-L1 expression on tumor cells (N=40)	Mean (sd)	19.45 (32)
	Median [IQR]	0 [0-27.5]
PD-L1 expression category (N=40)	High	10 (25)
	Moderate	7 (17)
	Negative	23 (58)
Percentage of CD73 tumor staining (N=40)	Mean (sd)	26.57 (29)
	Median [IQR]	20 [0-50]
CD73 tumor expression (N=35)	negative	11 (31)
	positive	24 (69)
Type of CD73 staining (N=24)	Complete	15 (62)
	Apical-lateral	9 (38)
CD73 TPS (N=35)	High (>50%)	4 (11)
	Low (≤50%)	31 (89)

CD73 Hscore	High (>150)	4 (11)
	Low (≤150)	31 (89)
CD73 expression on lymphocytes (N=35)	Negative	33 (94)
	Positive	2 (6)
CD73 expression on macrophages (N=35)	Negative	33 (94)
	Positive	2 (6)
CD73 expression on stromal cells (N=73)	Negative	44 (100)
	Positive	0 (0)
**Molecular data**		
EGFR mutation at baseline	Ex18_G719	1 (2)
	L858R	18 (41)
	del19	25 (57)
T790M during follow-up (N=24)	no	12 (50)
	yes	12 (50)
EGFR amplification status (N=41)	Amplification	30 (73)
	low trisomy	4 (10)
	low polysomy	7 (17)
