# Supplementary figures and images for: Exploring the Expression of CD73 in Lung Adenocarcinoma with EGFR Genomic Alterations
Source: Cancers (Basel). 2025 Mar 20;17(6):1034. doi: 10.3390/cancers17061034 (PMC11941413; doi:10.3390/cancers17061034)

## Slide 1
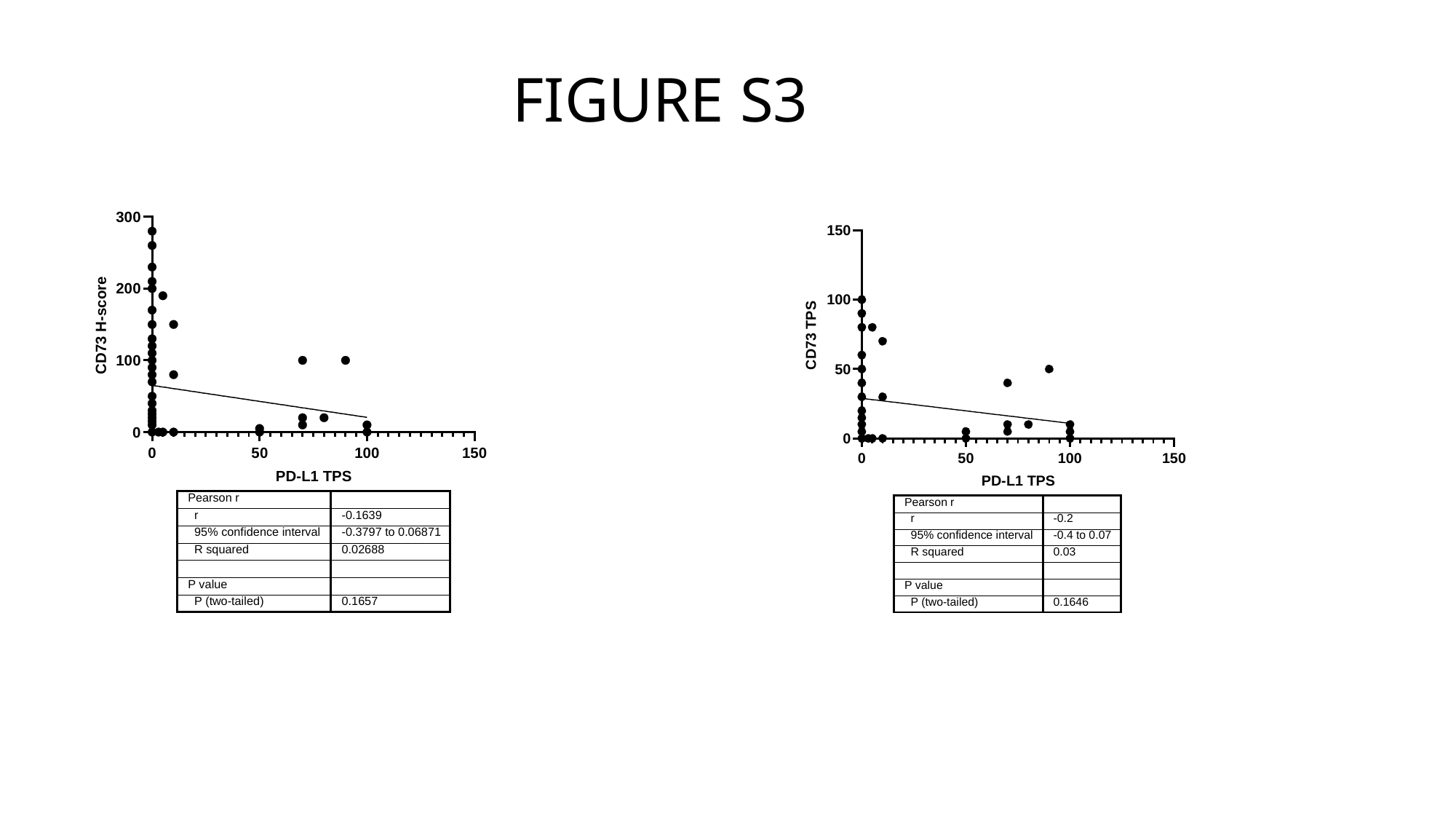

# FIGURE S3

Supplement: Supplementary file 1 [file cancers-17-01034-s001.zip › Figure S3.pptx]

## Slide 1
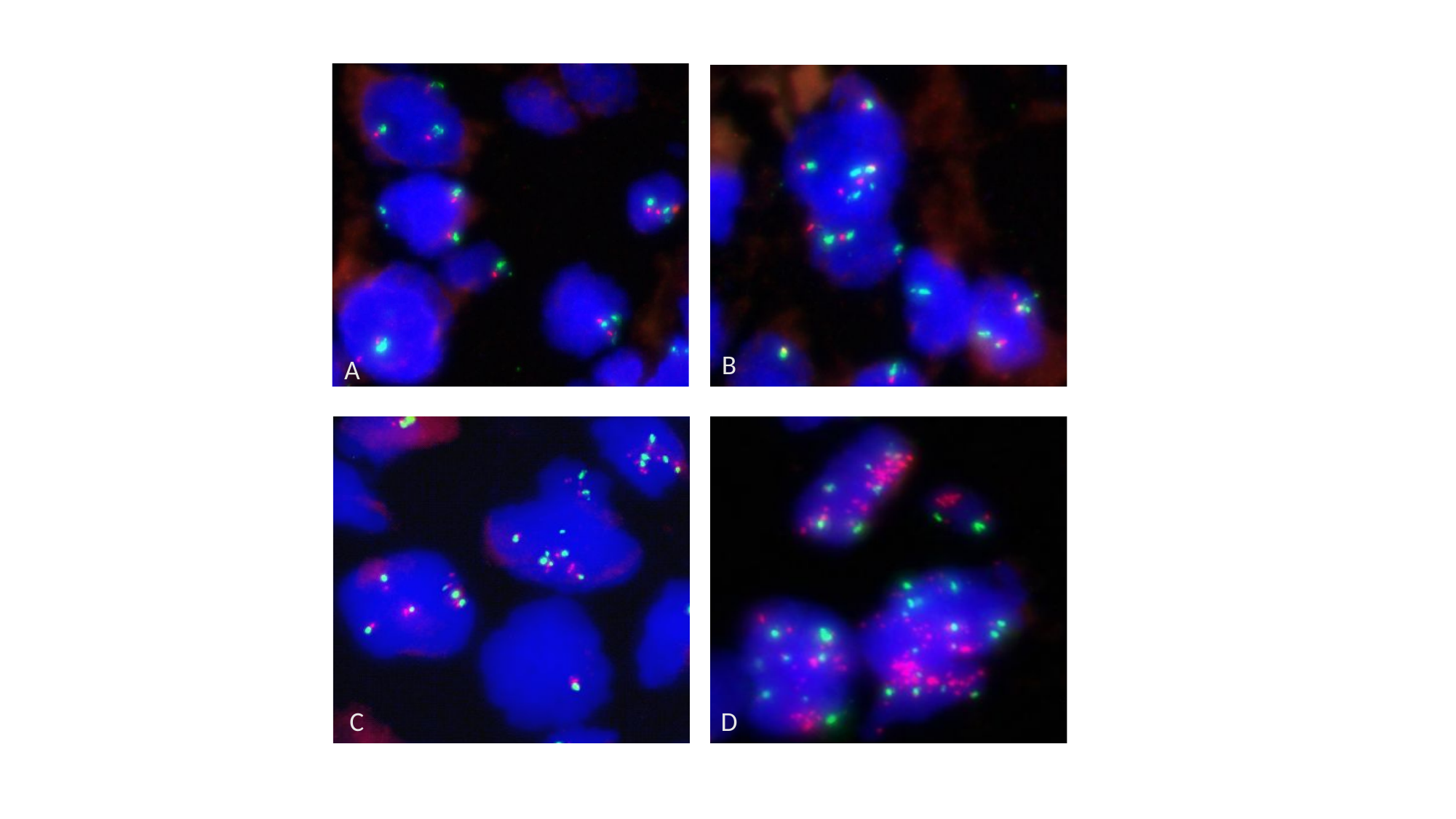

B
A
C
D

Supplement: Supplementary file 1 [file cancers-17-01034-s001.zip › Figure S4.pptx]

## Slide 1
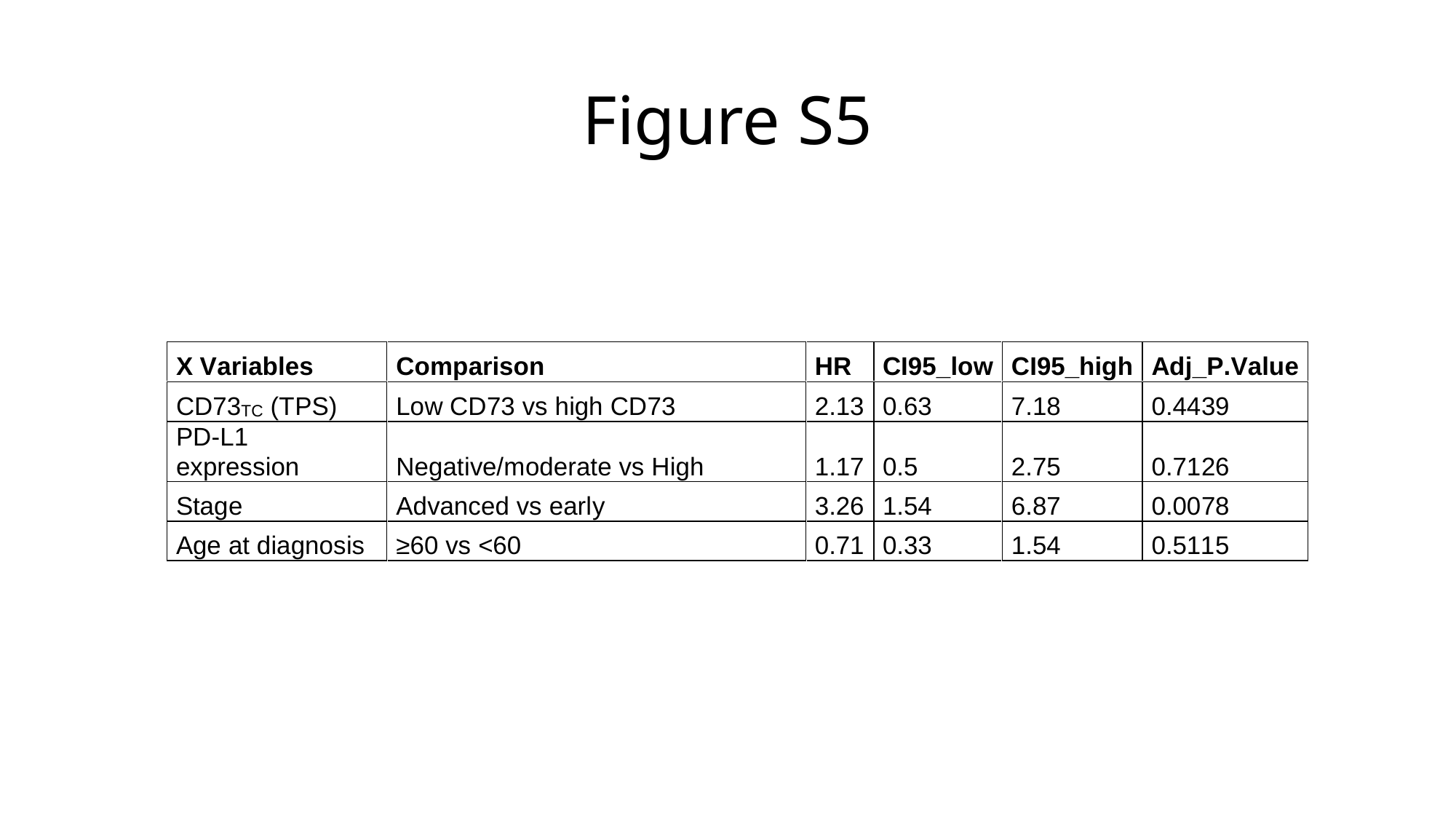

# Figure S5

Supplement: Supplementary file 1 [file cancers-17-01034-s001.zip › Figure S5.pptx]

## Slide 1
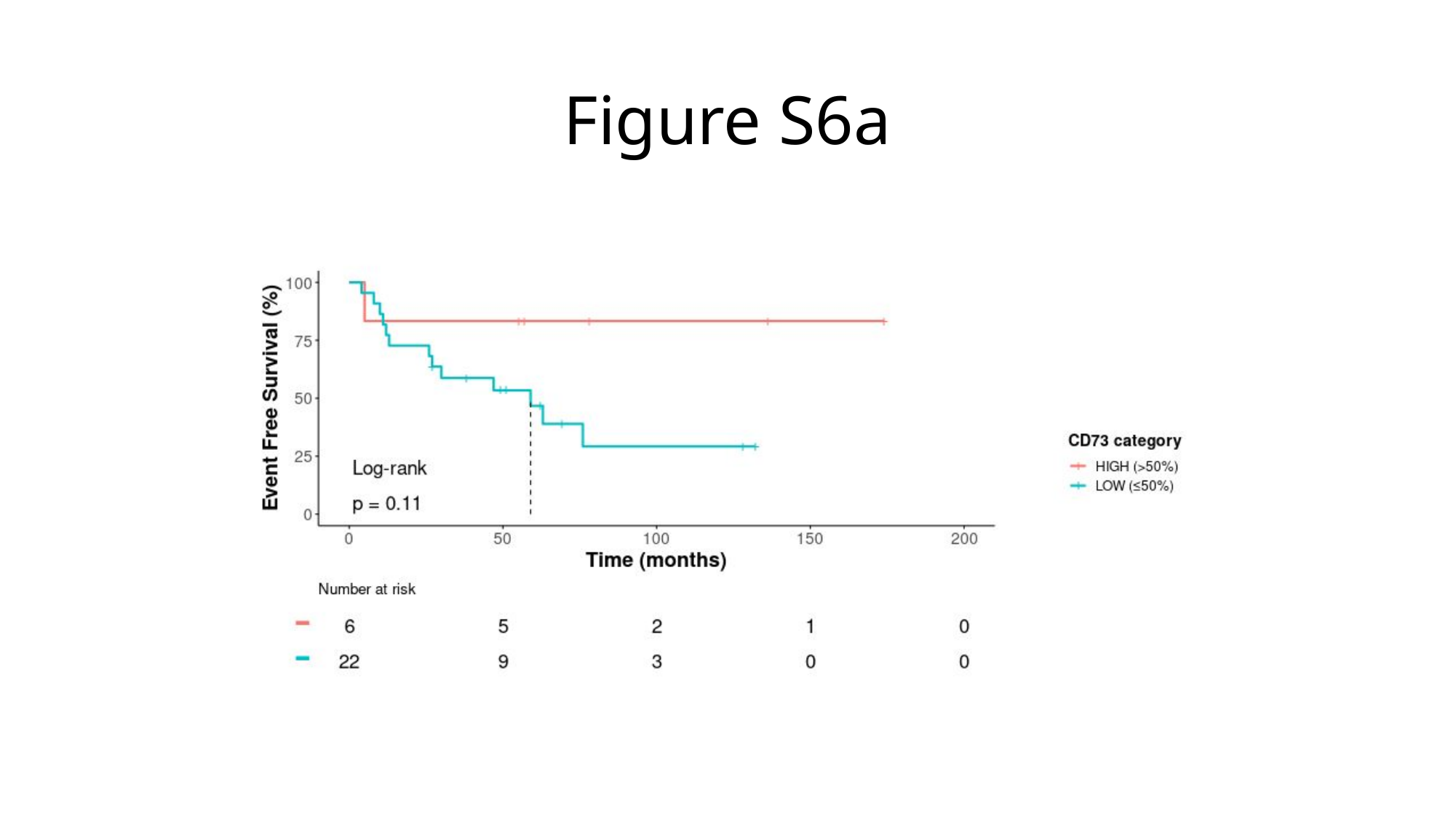

# Figure S6a

## Slide 2
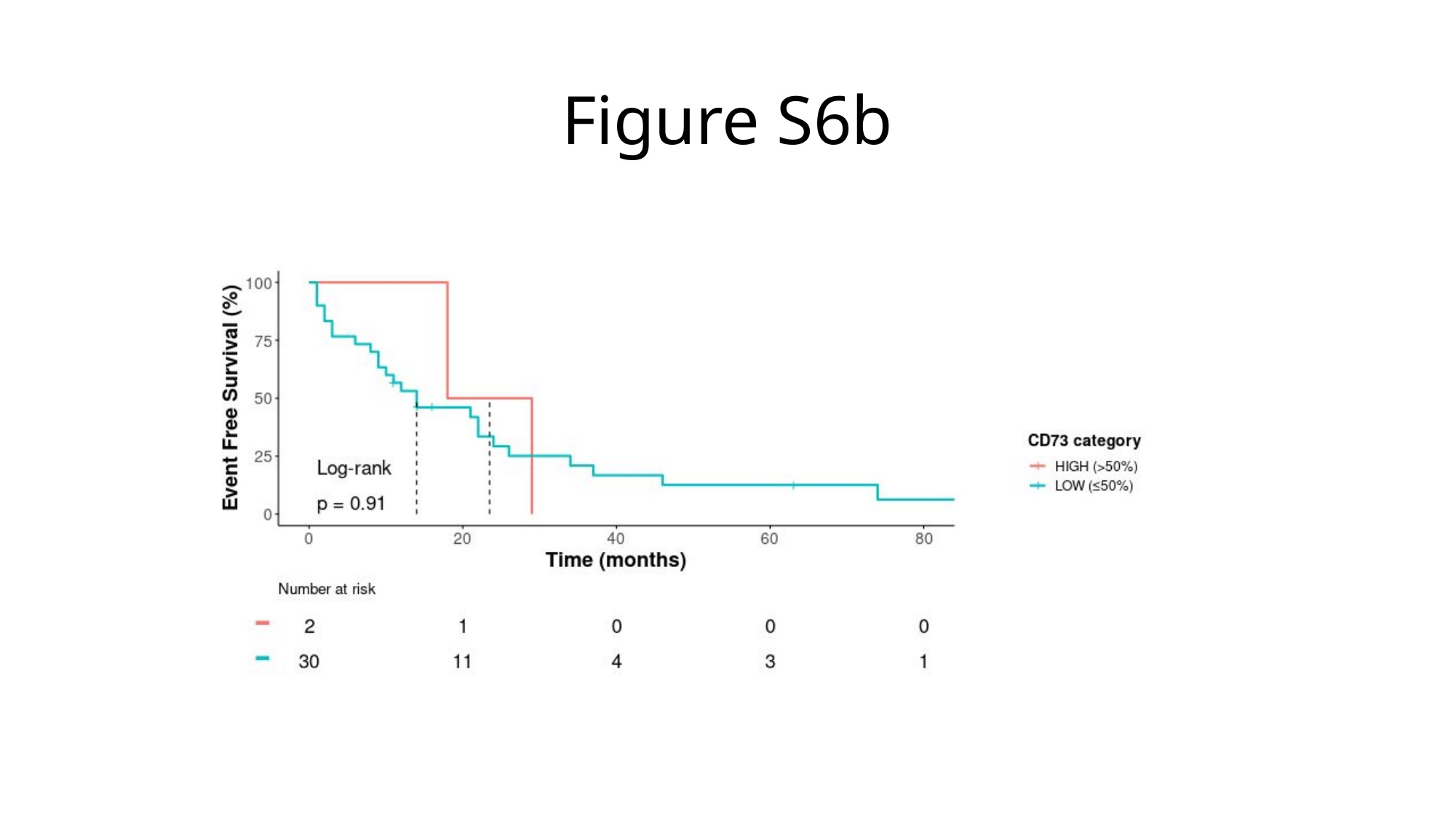

# Figure S6b

Supplement: Supplementary file 1 [file cancers-17-01034-s001.zip › Figure S6a-S6b.pptx]

## Slide 1
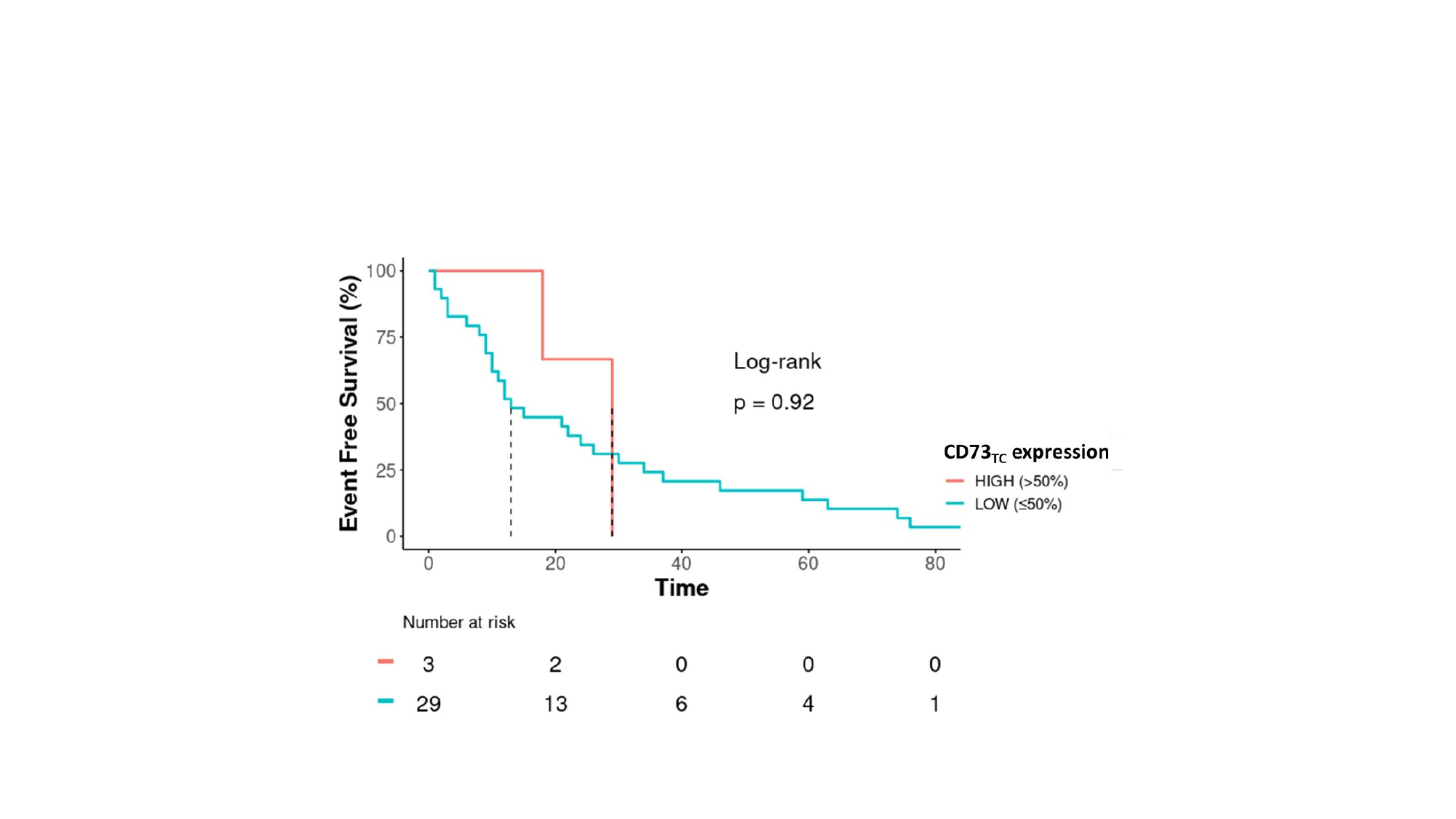

Supplement: Supplementary file 1 [file cancers-17-01034-s001.zip › Figure S7.pptx]
